# Trichina

**Published:** 1866-06

**Authors:** 


*Trichina.*—The aversion to pork, produced by the agitation of the subject of trichiniasis, has induced the Chicago Academy of Sciences to appoint a committee to investigate the subject, with a view to ascertain the exact state of facts, in order that the public may know whether the trichina exists in this region or not, and if it does, whether the consumers of pork can, in any way, be assured of safety in the use of this important staple. The present state of uncertainty in the public mind is injurious to various interests.

The committee commenced by obtaining specimens of muscle from about 1300 different hogs, from all parts of the West, fed in various ways, and raised in all sorts of circumstances. These specimens were distributed among eight of the best microscopists of this city, for examination. The result showed that trichina, beyond all doubt, exists here, and probably has existed through all time wherever hogs have lived. Among the 1300 animals, 28 were found to contain the parasite, which is rather more than 1 animal in 50, or 2 per cent. Of these, only 3 or 4 contained them in dangerous quantities. The committee reports that the statement that trichinæ can endure a boiling heat without death, is false and absurd. By actual experiment, they are found to be always killed by a temperature of 150° Fah. It is proved, also, that salting and smoking for 10 days is fatal to them. A case of trichiniasis is reported at Detroit, Mich., in the *Medical Review*, in which a woman, lately arrived from Germany, died, having eaten the parasites before her arrival here. Two cases were also reported in the *Buffalo Medical Journal*, two or three years since, but with the exception of these three, the committee cannot learn of any deaths clearly traceable to trichiniasis in this country. In Germany, where so many deaths have occurred, the poor people have a habit of eating a large amount of raw pork, in which state the parasite is taken alive into the stomach of the patient; but in this country, pork is universally well cooked, even by the most indigent. This accounts for the fact, that though trichinæ are doubtless as ancient and universally distributed as the hogs themselves, and Americans have always made pork a very prominent article of diet, yet the disease is almost unknown this side of the water.

The conclusion is, that pork in a raw state, or not thoroughly cooked, is a dangerous food; but, when well cooked to the centre, is as absolutely safe as any other article of diet. It is proper to add, that trichinæ are found in a variety of animals besides swine, and that the same rule, as to thorough cooking, ought to be applied to all, whether fish, flesh, or fowl. Not only is trichina found widely distributed, but also the germ of the tapeworm may be found in a variety of animals used for food. Beef and mutton are, probably, more free from parasitic dangers than any of the meats in common use.—*Chicago Medical Journal.*

